# Immunosuppressive effect of mesenchymal stem cell-derived exosomes on a concanavalin A-induced liver injury model

**DOI:** 10.1186/s41232-016-0030-5

**Published:** 2016-11-17

**Authors:** Ryo Tamura, Shinji Uemoto, Yasuhiko Tabata

**Affiliations:** 1 Department of Surgery, Graduate School of Medicine, Kyoto University, Kyoto, Japan; 2 Department of Biomaterials, Field of Tissue Engineering, Institute for Frontier Medical Sciences, Kyoto University, Kyoto, Japan

**Keywords:** Exosome, Mesenchymal stem cell, Concavanalin-A, Immune-induced liver injury, Nanovesicle, Transplantation

## Abstract

**Background:**

This study aimed to evaluate the effect of mesenchymal stem cell (MSC)-derived exosomes on an immune-induced liver injury model. MSCs show a unique function to modulate immune reaction although the molecular mechanisms are still under investigation. Exosomes are a nanoparticle containing microRNA and many ligands and are recognized as important factors secreted from MSC to express their function. This research is undertaken to evaluate the effect of MSC-derived exosome on concanavalin-A (con-A)-induced liver injury.

**Methods:**

Exosomes were collected from the supernatant of MSC from the bone marrow of C57B6 mice with ultracentrifugation. The collected exosomes or MSCs were injected intravenously into liver injury mice that had been prepared by the intravenous con-A injection. Liver and serum samples were collected 24 h later to evaluate the macro- and microscopic images, the alanine aminotransferase (ALT), and cytokine messenger RNA (mRNA) expression levels. Phenotypical change of non-parenchymal liver cells was also evaluated by flow cytometry. Liver localization of PKH26 after the injection of PKH26-labeled exosomes or MSCs was observed by microscope. Each result was statistically analyzed with Student’s *t* test.

**Results:**

PKH was observed in the liver after PKH-labeled exosomes were injected into mouse, whereas it was only observed in the lung in a mouse group receiving PKH-leveled MSC. There were decreases in ALT, liver necrotic areas, and the extent of apoptosis indicated by the single-stranded DNA index of groups that received multiple injections of MSC-derived exosomes, but an increase in the Ki-67 index. The mRNA expression of anti-inflammatory cytokines was enhanced. The number of Treg was increased among NPCs in a group receiving exosomes multiple times.

**Conclusions:**

Suppression of con-A-induced liver injury by injection of exosomes was observed as same extent as MSC. Considering the advantage of exosomes as its non-living nature and dosing adjustability over MSC, exosome will be one alternative of MSC transplantation.

## Background

For the success of organ transplantation, it is crucial to control organ rejection. Although the development of cyclosporine A and tacrolimus, which suppress interleukin (IL)-2 expression and T cell-mediated immune responses, was a breakthrough, their adverse effects are still a major problem in the long-term follow-up of transplant recipients. In particular, pediatric patients require long-term immune control, and development of a novel approach to control organ rejection is desired. Mesenchymal stem cells (MSCs) have the potential to self-renew, differentiate into multiple cellular lineages, and modulate immune properties [1–6]. It has been reported that MSC can be applied as promising immune modulators to minimize the use of immunosuppressants and decrease the occurrence of their adverse events [7–9]. MSC functions are mediated through the secretion of trophic factors in exosomes [10–12]. Exosomes are nanoparticles consisting of a lipid bilayer, which transport various molecules for intercellular communication [13, 14]. MSC-derived exosomes have been reported as a non-cellular alternative to MSC for transplantation therapy [15–17]. Exosomes are a non-cellular resource and therefore free from the potential to differentiate into unintended cellular lineages, which is a major problem of MSC transplantation [18]. Exosomes can be injected multiple times, and their characteristics make the therapeutic use of exosomes more flexible. The advantages of exosomes provide a therapeutic benefit for younger patients who require more adjustable and sustainable treatments rather than one-time fixed dosing such as MSC transplantation.

In this study, we investigated the suppressive effect of exosomes on an immune-induced liver injury model. The liver injury was induced by injection of concanavalin A (con-A), a lectin derived from jack beans. This type of liver injury is mediated through activation of the adaptive immune system including natural killer and Kupffer cells, which is employed as a method to evaluate the induction liver tolerance [19–21]. The suppressive effects of MSC-derived exosomes and the MSCs in this injury model were evaluated by the level of plasma alanine aminotransferase (ALT), histopathological examinations, the messenger RNA (mRNA) expression of pro- and anti-inflammatory cytokines, and the population alteration of regulatory T cells (Treg) among non-parenchymal liver cells (NPCs). Additionally, we performed in vivo tracing with fluorescently labeled exosomes and MSCs in liver tissues.

## Methods

### Animals and cultivation of MSCs and fibroblasts

Male C57B6 mice were maintained under specific pathogen-free conditions at the animal facility of Kyoto University. All animal experiments were carried out in accordance with the procedures approved by Animal Experimentation Committee of Institute for Frontier Medical Sciences (approval number #F173). MSCs were harvested from the bone marrow of 8-week-old male C57B6 mice according to a previous report [22] with slight modification. In brief, bone marrow cells were collected from the femurs and tibias of mice by flushing with fresh complete Dulbecco’s modified Eagle’s medium (DMEM) containing 15% endotoxin-free fetal bovine serum (FBS) and 1% penicillin-streptomycin. The FBS had been centrifuged at 100,000*g* for 3 h at 4 °C to deplete vesicles and protein aggregates before use [23]. The bone marrow cells were cultured in complete DMEM for 3 h before the first medium change. Thereafter, the medium was replaced every 8 h for the next 24 h and then every 12 h for the next 48 h. The cells were washed after 72 h of culture, and the medium was replaced every 3–4 days. Passaging was carried out at 2 weeks after the initiation of culture and repeated every 7 days. MSCs and the culture supernatants of cells passaged one to three times were used for the following experiments. Harvesting and culture of fibroblasts from the back skin of 4-week-old male C57B6 mice were carried out according to a previous report [24]. Culture supernatants of fibroblasts passaged one to four times were used to collect exosomes.

### Characterization of MSC and NPC

The differentiation capacity of MSCs for adipocytes, osteocytes, and chondrocytes was evaluated using StemPro® Adipogenesis, Osteogenesis, and Chondrogenesis Differentiation Kits (Life Technologies, Carisbad, CA, USA) according to the manufacturer’s protocols. Histochemical staining with oil red O, alizarin red, and alcian blue was performed as a measure of adipogenesis, osteogenesis, and chondrogenesis, respectively, according to the manufacturers’ protocols.

NPC from harvested liver samples were also collected according to a previously reported procedure [21]. Passage 3 MSCs were employed. Their preparation and staining were carried out based on a previous report [21]. The monoclonal antibodies (mAbs) are listed in Table 1. Intracellular staining of FoxP3 in NPCs was carried out using an anti-mouse/rat FoxP3 Staining Set (eBioscience, San Diego, CA, USA) according to the manufacturer’s protocol. A FACS CANTO II and FACS Diva software (BD Bioscience, Franklin Lakes, NJ, USA) were used for analysis.

**Table 1:** Antibody used in flow cytometry or immunofluorescent study

Antibody	Source	Catalogue#	Clone
Anti-CD16/32	Biolegend (San Diego, CA, USA)	1013009	93
FITC-labeled anti-CD3	Biolegend	B134497	17A2
FITC-labeled anti-CD9	eBioscience	11-0091-81	KMC8
FITC-labeled anti-CD31	Biolegend	B112061	MEC13.3
FITC-labeled anti-FoxP3	eBioscience	11-5775-80	FJK-16s
PE-labeled anti-CD25	eBioscience	12-0251-81	PC61.5
PE-labeled anti-CD44	BD Bioscience	553134	IM7
PE-labeled anti-CD63	Cosmo Bio Co. (Tokyo, Japan)	143904	NVG-2
PE-labeled anti-CD117	eBioscience	12-1171	2B8
PE-Cy5-labeled anti-Sca1	eBioscience	25-5981-82	D7
PerCP-labeled anti-B220	Biolegend	103234	RA3-6B2
eFluor®660-labeled anti-CD34	eBioscience	560238	RAM34
APC-eFluor®780-labeled anti-CD45	eBioscience	47-0451-82	30-F11
PE-Cy7-labeled anti-CD4	BD Bioscience	552775	RM4-5
Purified anti-F4/80	Biolegend	123109	BM8
Purified anti-CD81	eBioscience	14-0811-81	EAT2
Alexa fluor 647 goat anti-rat IgG	Thermo Fisher Scientific (Waltham, MA, USA)	A21247	

### Collection of exosomes

Exosomes were purified from the culture supernatants of MSCs and fibroblasts. Collection was carried out based on a previous report [14] with slight modification. The culture supernatants of MSCs and fibroblasts were centrifuged at 2000*g* and 10,000*g* for 20 and 30 min, respectively, to deplete cell debris, and the final supernatants were filtered through 0.45-μm pore filters [25, 26]. Then, the filtrate was ultracentrifuged to pellet the exosomes. The total amount of collected exosomes was determined by measuring the protein concentration with a BCA assay kit (Thermo Fisher Scientific) according to the manufacturer’s protocol.

### Characterization of exosomes collected

The size distribution of the exosomes collected was determined using a qNano (Izon, Christchurch, New Zealand) according to the manufacturer’s protocol. Flow cytometric analysis of exosomes was carried out according to previous reports [25, 27]. In brief, 10 μg exosomes was reacted with 20 μl aldehyde/sulfate beads (4 μm in diameter, Life Technologies) overnight at 4 °C. After washing with PBS containing 0.2% FBS and 0.05% sodium azide, the beads were incubated with anti-CD9 and -CD63 mAbs (Table 1) and then analyzed. The morphology of exosomes was observed by transmission electron microscopy (TEM) as reported previously [28].

### Fluorescent labeling of exosomes and MSCs with PKH26

Fluorescent labeling of exosomes was carried out according to a previously reported procedure [29]. In brief, 100 μg exosomes was reacted with 4 μl PKH26 dye from a PKH26 staining kit (Sigma-Aldrich, St. Louis, MO, USA). Fluorescent labeling of MSC was carried out according to the manufacturer’s protocol.

### Preparation of the mouse liver injury model and administration of exosomes and MSCs

con-A (Cosmo Bio Co.) in PBS was injected intravenously (15 mg/kg body weight). Immediately after the injection, 0.1 ml PBS containing 10 μg MSC- or fibroblast-derived exosomes or 1 × 10^6^ MSC suspended in 0.5 ml PBS was injected. Specific groups received multiple injections of MSC- or fibroblast-derived exosomes at 0, 8, and 16 h after con-A injection. For tracing experiments, the same amount of PKH26-labeled exosomes or number of MSCs was employed. Each group included five mice.

### Sample collection and immunohistochemical staining of liver specimens

Liver and blood samples were harvested at 24 h after the beginning of in vivo experiments. For exosome tracing, the liver was harvested at 4 h after the initiation of experiments. The plasma ALT level was measured using a standard clinical automatic analyzer. Hematoxylin-eosin, Ki-67, and single-stranded DNA (ssDNA) staining was carried out using tissue specimens that were fixed in 10% buffered formalin (Wako Pure Chemical Industries Ltd, Osaka, Japan) overnight and then embedded in paraffin. Each type of staining was carried out with a standard protocol. Immunofluorescence staining was carried out using frozen sections prepared according to a previous report [28]. Primary and secondary antibodies used at a dilution of 1:1000 are shown in Table 1. Observations and imaging analysis were carried out with a Keyence BZ-X710 fluorescence microscope and BZ-X analyzer.

### mRNA expression analysis of inflammatory cytokines

Total RNA was purified from liver tissue using TRIzol® Reagent (Thermo Fisher Scientific) according to the manufacturer’s protocol. cDNA was generated from 1 μg purified RNA using a SuperScript VILO cDNA synthesis kit (Thermo Fisher Scientific). The cDNA was analyzed using the Power SYBR® Green quantitative fluorescent PCR method (Thermo Fisher Scientific) with a 7500 Real-time PCR system (Applied Biosystems, Foster city, CA, USA). The primers are summarized in Table 2. The following PCR conditions were used: 95 °C for 10 min, followed by 40 cycles of 94 °C for 15 s and 60 °C for 30 s. Glyceraldehyde-3-phosphate dehydrogenase was used as a housekeeping gene. Fold induction was calculated using the C_t_ method, ∂∂C_t_ = (C_t_^target^ − C_t_^housekeeping^)^infected^ − (C_t_^target^ − C_t_^housekeeping^)^uninfected^, and the final data were derived from 2^−∂∂Ct^.

**Table 2:** Primers used for quantitative real-time PCR

mRNA	Forward	Reverse
IL1b	5′-TTCCCCAGGGCATGTTAAGG-3′	5′-TTCTTGTGACCCTGAGCGAC-3′
IL2	5′-CCTGAAACTCCCCAGGATGC-3′	5′-TCAAATCCAGAACATGCCGC-3′
IL4	5′-GTAGGGCTTCCAAGGTGCTT-3′	5′-GGCATCGAAAAGCCCGAAAG-3′
IL10	5′-CAGAGCCACATGCTCCTAGA-3′	5′-GTCCAGCTGGTCCTTTGTTT-3′
HGF	5′-CACCCCTTGGGAGTATTGTG-3′	5′-GGGACATCAGTCTCATTCACAG-3′
TNFα	5′-TCTTCTCATTCCTGCTTGTGG-3′	5′-GGTCTGGGCCATAGAACTGA-3′
TGFβ	5′-TGGAGCAACATGTGGAACTC-3′	5′-CAGCAGCCGGTTACCAAG-3′
INFγ	5′-GGAGGAACTGGCAAAAGGAT-3′	5′-TTCAAGACTTCAAAGAGTCTGAGG-3′
GAPDH	5′-TGTTGAAGTCACAGGAGACAACCT-3′	5′-AACCTGCCAAGTATGATGACATCA-3′

*IL* interleukin, *HGF* hepatocyte growth factor, *TNFα* tumor necrosis factor-α, *TGFβ* transforming growth factor-β, *INFγ* interferon-γ, *GAPDH* glyceraldehyde-3-phosphate dehydrogenase

### Statistical analysis

Data are presented as means ± standard error of the mean. Comparisons between two groups were carried out using the two-tailed Student’s *t* test. Differences were considered significant at *p* < 0.05.

## Results

### Characterization of MSCs and exosomes

**Fig. 1:**
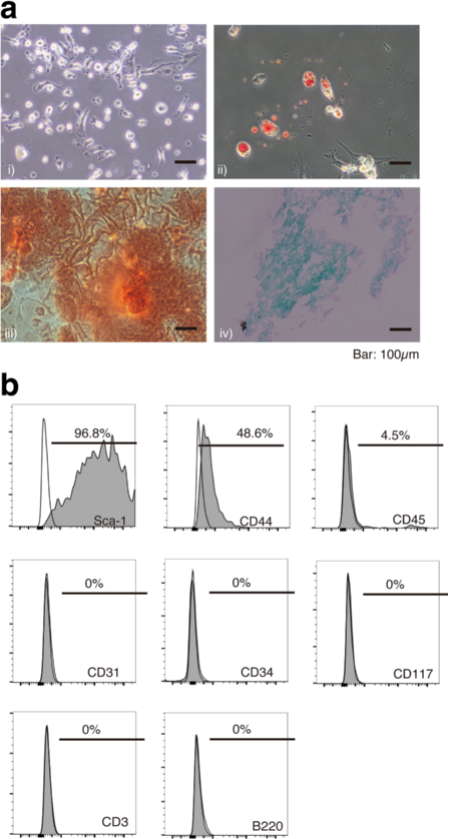
Characterization of MSCs and exosomes. **a** Differentiation of MSCs into three cellular lineages. *i)* Microscopy images of MSCs, *ii)* MSCs stained with oil red O at 7 days after adipogenic differentiation culture, *iii)* MSCs stained with alizarin red staining at 30 days after osteogenic differentiation culture, and *iv)* MSCs stained with alcian blue staining at 18 days after chondrogenic differentiation culture. **b** Flow cytometric analysis of MSCs

**Fig. 2:**
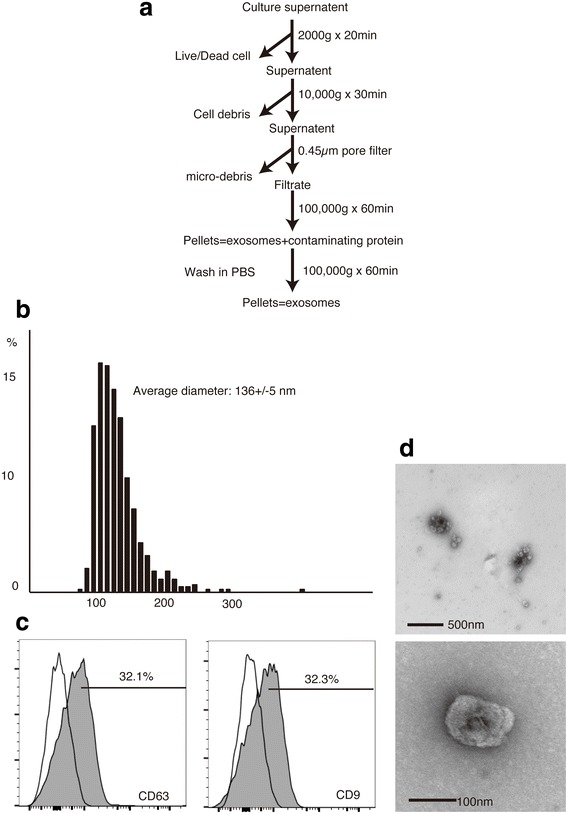
Characterization of exosomes collected. **a** Collection procedure for exosomes. **b** Size distribution of exosomes. **c** TEM images of exosomes. **d** Surface markers of exosomes

Bone marrow-derived cells had a small cell body with numerous long and thin cell processes (Fig. 1a). Immunohistochemical staining demonstrated their capacity for adipogenic, osteogenic, and chondrogenic differentiation. Passage 3 MSCs expressed undifferentiated MSC antigens CD44 and Sca1. CD31, CD34, CD45, CD117, CD3, and B220 antigens for mesenchymal progenitor cells and differentiated lymphocytes/leukocytes were scarcely detected (Fig. 1b). The average size of the collected exosomes was 135 nm (Fig. 2b). The percentage of exosomes with CD9 and CD63 surface markers was 20–30% (Fig. 2c). Exosomes showed a typical cup shape of 100–150 nm (Fig. 2d).

### In vivo accumulation of PKH26-labeled exosomes and MSCs and co-localization of PKH and CD81

**Fig. 3:**
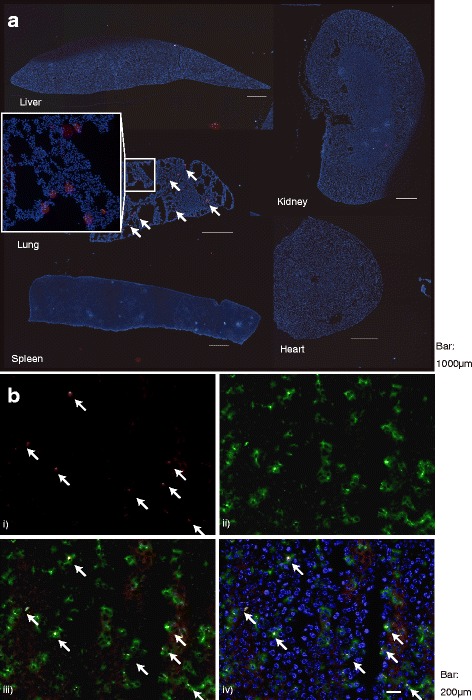
In vivo tracing of PKH26 fluorescent dye-labeled MSCs and exosomes. **a** Localization of PKH26 dye after the injection of PKH26-labeled MSC (*arrow*). *White box* showed the magnified image of the place where PKH was observed in the lung. **b** Presence of PKH26 dye in the liver specimen and their co-localization with FITC-stained F4/80-positive cells after the injection of PKH26-labeled exosomes (*arrow*). *i)* PKH26 after the injection of PKH26 labeled-exosomes, *ii)* FITC-stained F4/80-positive cells, *iii)* merged image of FITC-stained F4/80-positive cells and PKH-labeled exosomes, and *iv)* merged image of PKH26, F4/80-positive cells, PKH26, and DAPI

**Fig. 4:**
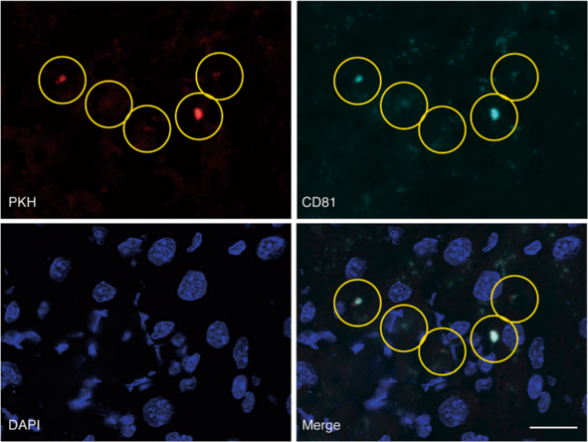
Co-localization of PKH and CD81 on the liver specimen. Co-localization of PKH and CD81, a common surface marker of exosomes, was shown in the *yellow circles* in each picture of PKH, CD81, and their merged image with DAPI staining

PKH26 was observed as bright red spots. PKH26 was not only observed in the injured liver whereas it was mainly observed in the lung (Fig. 3a). On the other hand, PKH26 was detected in injured liver tissue after the injection of PKH26-labeled exosomes (Fig. 3b). When intrahepatic sinusoid resident macrophages were visualized by staining the typical marker F4/80, most PKH26 red spots were co-localized with F4/80-positive cells. A co-localization of PKH and CD81, a common surface marker of exosomes, was also observed in the liver specimen (Fig. 4).

### In vivo effect of exosomes derived from MSCs and fibroblasts

**Fig. 5:**
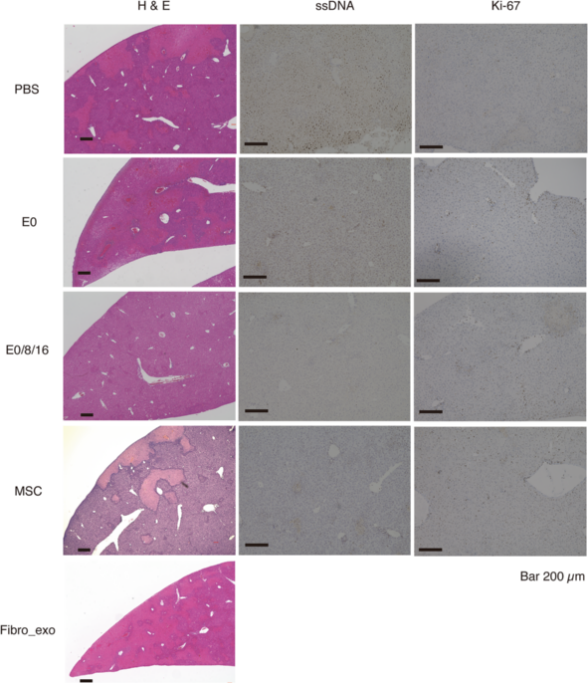
Histological findings of the liver at in vivo experiment with exosomes derived from MSCs and fibroblasts. Hematoxylin-eosin, Ki-67, and ssDNA staining of the liver of mice received intravenous injections of PBS only (*PBS*), MSC-derived exosomes once (*E0*), MSC-derived exosomes three times (*E0/8/16*), MSCs (*MSC*), or fibroblast-derived exosomes three times (*Fibro_exo*)

**Fig. 6:**
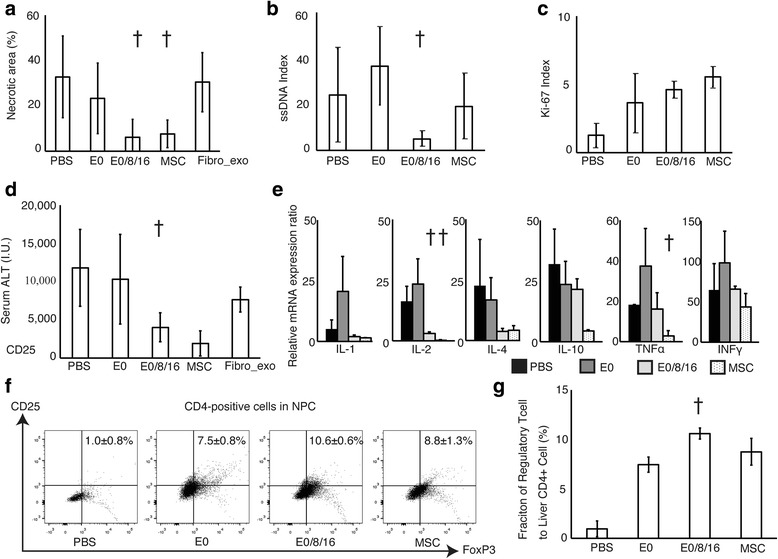
In vivo effect of exosomes derived from MSC. mRNA expression of pro- and anti-inflammatory cytokines and flow cytometric analysis of Treg among NPCs. **a**–**c** Percentage of necrotic areas in the injured liver evaluated with hematoxylin-eosin staining (**a**), ssDNA index (**b**), and Ki-67 index (**c**) of mice received intravenous injections of PBS only (*PBS*), MSC-derived exosomes once (*E0*), MSC-derived exosomes three times (*E0/8/16*), or MSCs (*MSC*). **d** Plasma ALT levels. **e** mRNA expression ratios of pro- and anti-inflammatory cytokines. **f**, **g** Dot plots (**f**) and percentage (**g**) of Treg to CD4-positive percentage of Treg to CD4-positive cells among NPCs. ^†^ *p* < 0.05 vs. the group that received a single injection of exosomes

The group that received three injections of MSC-derived exosomes showed significant decreases in necrotic areas (Figs. 5 and 6a) and ssDNA index (Fig. 6b) and increase of Ki-67 (Fig. 6c) index, respectively. The percentage ssDNA index was significantly decreased in the group that received three injections of MSC-derived exosomes (Fig. 6b), whereas the percentage of Ki-67-positive cells was significantly increased in groups that received exosome or MSC injections (Fig. 6c). Plasma ALT level of same group was suppressed at similar extents to those in the MSC-injected treatment group (Fig. 6d). In the group that received three injections of fibroblast-derived exosomes, the values tended to decrease but were not as low as those in the group received three injections of MSC-derived exosomes.

### mRNA expression of pro- and anti-inflammatory cytokines and flow cytometric analysis of NPCs

Messenger RNA expression of the pro-inflammatory cytokine IL-2 was significantly decreased in groups that received three injections of exosomes or MSCs (Fig. 6e), while that of transforming growth factor β (TGFβ) and hepatocyte growth factor (HGF) was increased in the group that received three injections of exosomes. The percentage of Treg to CD4-positive cells among NPCs was increased significantly by treatment with exosomes or MSCs (Fig. 6f, g). The group that received three injections of exosomes had the most significant increase in the number of Treg. A statistical difference was observed between the group received three injections of exosomes and the group that received a single injection of same exosomes.

## Discussion

The present study demonstrates the promising efficacy of exosomes on con-A-induced liver injury model in mouse. Exosomes are interesting and usable biological nanovesicles that have the ability to carry and transport many substances into other cells [13, 14]. They are regarded as a component of the paracrine substances of MSC and have been applied as therapeutics in animal models [12, 15]. MSCs were identified as plastic-adherent, bone marrow-derived cells with a spindle shape, and their immune-modulating property has been applied to cell transplantation therapy for various diseases [1, 6, 7, 30]. Multi-lineage differentiation capacity of MSC used in this study was confirmed by the staining for adipogenic, osteogenic, and chondrogenic cellular differentiations (Fig. 1a). Furthermore, flow cytometry showed the presence of typical MSC markers such as Sca-1 and CD44 whereas absence of major lineage differentiation markers such as CD3, B220, and CD45 (Fig. 1b). Exosomes was collected by ultracentrifugation procedure without density gradient for purification (Fig. 2a). The average size of exosomes collected was approximately 135 nm in diameter (Fig. 2b). Exosomes are often described as extracellular vesicles of smaller than 100 nm in diameter [31]. Based on the results of diameter distribution of exosomes collected by qNano system and TEM image (Fig. 2b, d), it was possible that the exosomes collected formed aggregates in the collection process as reported [32].

In vivo bio-distribution of MSC after the intravenous injection was still under investigation [33]. In our study, PKH that was used for labeling of MSC before intravenous injection of MSC was only observed in the lung as reported previously (Fig. 3a) [33]. MSC used in this study showed its suppressive effect on con-A-induced liver injury model while it was not observed in the injured liver, and it is possible to surmise some secreting factors from MSC played an important role in the expression of suppressive effect of MSC. At the same time, PKH used for labeling of exosomes in this study was observed in the injured liver (Fig. 3b). In this in vivo tracing study, most of them were co-localized with F4/80-positive cells that correspond to Kupffer cells [34]. Kupffer cell is specialized macrophages in the liver and plays an important role in engulfing of exosomes [35–37]. It also reported to be involved in the initiation and suppression of con-A-induced liver injury through the induction of natural killer and Treg [34, 38–40].

At the same time, co-localization of PHK with CD81, a common surface marker of exosomes, was observed in the injured liver specimen (Fig. 4). In this experiment, PKH was employed to label the surface of MSCs which were captured in the lung after intravenous injection. The presence of PKH in the liver and its co-localization with CD81 implied the releasing factors from MSCs in the lung reached to the liver specimen, and they possessed one of the unique surface markers of exosomes.

Multiple injections of MSC-derived exosomes exerted a significant effect to suppress the liver injury in terms of necrotic areas and ALT levels, which was similar to the MSC (Figs. 5 and 6a, d). However, fibroblast-derived exosomes did not exert a similar effect. It has been reported that the contents of exosomes and their functions are completely different depending on the character of the cells that secreted them [41]. Fibroblasts are not involved in the liver injury model used in this study, which may be the reason why only exosomes derived from MSC exerted the suppressive effect [42]. Single-stranded DNA is a marker of apoptotic cells, and a decrease in the ssDNA index was only observed in the group that received three injections of exosomes (Fig. 6b). At the same time, the number of Ki-67-positive cells was increased in groups that received injections of exosomes or MSCs (Fig. 6c). Ki-67 expression tended to increase after severe tissue damage. It is possible that the increase in Ki-67 expression of the group that received the single injection of exosomes was due to severe inflammation. However, the group that received three injections of exosomes or MSCs showed an increase in Ki-67 expression, even though the liver necrosis of both groups appeared to be less severe (Figs. 5 and 6a, c). This result was experimentally supported by the enhanced tissue regeneration in both groups expressed by a result of Ki-67 index and mRNAs of several cytokines (Fig. 6c, e). [43]. The mRNA expression of TGFβ and HGF, which are mainly involved in liver regeneration, also increased in the group that received three injections of exosomes (Fig. 6e) [44]. Considering the suppression of inflammation, these results imply that the con-A-induced liver injury was suppressed through decreased induction of hepatocyte apoptosis and enhancement of tissue regeneration.

In this study, it is interesting that only the group that received the single injection of exosomes showed enhanced expression of inflammatory cytokine mRNAs, even comparing a group receiving PBS only (Fig. 6e). Generally, MSC is reported to support the biological functions of polymorphonuclear cells and macrophages and consequently contribute to the maintenance of inflammation in the early phase, whereas it acts as an anti-suppressant and induces tissue regeneration in late inflammatory phase [11, 45, 46]. In this study, single injection of exosomes derived from MSC was carried out at the acute phase of liver inflammation. Exosomes are reported to have very short half-life in bloodstream [28]. Therefore, exosomes injected in this period would support and enhance the effect of polymorphonuclear cells and macrophages in this stage and consequently augment the inflammation in the liver. On the other hand, mice group received three times injection of exosomes showed suppression of liver injury similar extent to that of MSCs (Fig. 6a–d). In this study, the total amount of exosomes used in the three injections was approximately the same as that of exosomes collected from the culture supernatant of 1 × 10^6^ MSCs. This multiple injection strategy may mimic the continuous release of paracrine factors from MSCs and induce tissue regeneration.

An increase in the number of Treg was observed in groups that received injections of exosomes or MSCs, and the highest percentage was observed in the group that received three injections of exosomes (Fig. 6f, g). It has been reported that Treg are required to induce immune tolerance in this liver injury model [39, 40]. In addition, MSC-derived exosomes play an important role in the induction of Treg, which is observed in tissue regeneration governed by MSC [47, 48]. Taken together, it is highly conceivable that the injection of exosomes contributed to the induction of Treg in the injured liver and the subsequent amelioration of injury and enhancement of regeneration. The con-A-induced liver injury model is used to evaluate the tolerogenic effect of therapeutic molecules [21]. Therefore, we can say with certainty that the suppressive effect of MSC-derived exosomes shown in this study implies their immune-modulating property.

MSC-derived exosomes have been reported as a non-cellular alternative to MSC for transplantation therapy [15–17]. MSC transplantation is considered to be a promising adjunctive therapy to minimize the required amount of current immunosuppressants and to reduce their adverse effects [9]. However, unintended differentiation of transplanted cells has been reported, which is a potentially harmful complication of this therapy [18]. Exosomes are a non-cellular resource and therefore free from such problems. Furthermore, it is possible to control the dose, frequency, and timing of their administration more flexibly. Pediatric recipients of organ transplantation require long-term immune control, and it is strongly desired to develop a sustainable and adjustable treatment free from any adverse effects. The use of MSC-derived exosomes has major advantages to meet these requirements and may become a promising non-cellular alternative to MSCs for transplantation therapy.

## Conclusions

We demonstrated the suppressive effect of exosomes derived from MSCs on an immune-mediated liver injury model. MSC-derived exosomes have some advantages over MSCs and might become a promising alternative to MSCs for transplantation therapy.

## Abbreviations

ALT: Alanine aminotransferase
con-A: Concanavalin-A
DMEM: Dulbecco’s modified Eagle’s medium
FBS: Fetal bovine serum
GAPDH: Glyceraldehyde-3-phosphate dehydrogenase
HGF: Hepatocyte growth factor
IL: Interleukin
INFγ: Interferon-γ
mAbs: Monoclonal antibodies
mRNAs: Messenger RNAs
MSCs: Mesenchymal stem cells
NPC: Non-parenchymal liver cells
ssDNA: Single-stranded DNA
TEM: Transmission electron microscopy
TGFβ: Transforming growth factor-β
TNFα: Tumor necrosis factor-α
Treg: Regulatory T cells
